# Inhibition of Extracellular Cathepsin D Reduces Hepatic Lipid Accumulation and Leads to Mild Changes in Inflammationin NASH Mice

**DOI:** 10.3389/fimmu.2021.675535

**Published:** 2021-07-16

**Authors:** Tulasi Yadati, Tom Houben, Albert Bitorina, Yvonne Oligschlaeger, Marion J. Gijbels, Ronny Mohren, Dieter Lütjohann, Princy Khurana, Sandeep Goyal, Aditya Kulkarni, Jan Theys, Berta Cillero-Pastor, Ronit Shiri-Sverdlov

**Affiliations:** ^1^ Department of Molecular Genetics, School of Nutrition and Translational Research in Metabolism (NUTRIM), Maastricht University, Maastricht, Netherlands; ^2^ Department of Medical Biochemistry, Experimental Vascular Biology, Amsterdam UMC, University of Amsterdam, Amsterdam, Netherlands; ^3^ Department of Pathology CARIM, Cardiovascular Research Institute Maastricht, GROW-School for Oncology and Developmental Biology, Maastricht University, Maastricht, Netherlands; ^4^ Maastricht Multimodal Molecular Imaging Institute (M4I), Division of Imaging Mass Spectrometry, Maastricht University, Maastricht, Netherlands; ^5^ Institute of Clinical Chemistry and Clinical Pharmacology, University of Bonn, Bonn, Germany; ^6^ Aten Porus Lifesciences Pvt Ltd, Bengaluru, India; ^7^ The M-Lab, Department of Precision Medicine, GROW - School for Oncology, Maastricht University, Maastricht, Netherlands

**Keywords:** NASH, lysosomal enzymes, lipoprotein metabolism, inflammation, extracellular cathepsin D, small-compound inhibitors

## Abstract

**Background & Aims:**

The lysosomal enzyme, cathepsin D (CTSD) has been implicated in the pathogenesis of non-alcoholic steatohepatitis (NASH), a disease characterised by hepatic steatosis and inflammation. We have previously demonstrated that specific inhibition of the extracellular CTSD leads to improved metabolic features in Sprague-Dawley rats with steatosis. However, the individual roles of extracellular and intracellular CTSD in NASH are not yet known. In the current study, we evaluated the underlying mechanisms of extracellular and intracellular CTSD fractions in NASH-related metabolic inflammation using specific small-molecule inhibitors.

**Methods:**

Low-density lipoprotein receptor knock out (*Ldlr-/-*) mice were fed a high-fat, high cholesterol (HFC) diet for ten weeks to induce NASH. Further, to investigate the effects of CTSD inhibition, mice were injected either with an intracellular (GA-12) or extracellular (CTD-002) CTSD inhibitor or vehicle control at doses of 50 mg/kg body weight subcutaneously once in two days for ten weeks.

**Results:**

*Ldlr-/-* mice treated with extracellular CTSD inhibitor showed reduced hepatic lipid accumulation and an associated increase in faecal bile acid levels as compared to intracellular CTSD inhibitor-treated mice. Furthermore, in contrast to intracellular CTSD inhibition, extracellular CTSD inhibition switched the systemic immune status of the mice to an anti-inflammatory profile. In line, label-free mass spectrometry-based proteomics revealed that extra- and intracellular CTSD fractions modulate proteins belonging to distinct metabolic pathways.

**Conclusion:**

We have provided clinically translatable evidence that extracellular CTSD inhibition shows some beneficial metabolic and systemic inflammatory effects which are distinct from intracellular CTSD inhibition. Considering that intracellular CTSD inhibition is involved in essential physiological processes, specific inhibitors capable of blocking extracellular CTSD activity, can be promising and safe NASH drugs.

## Introduction

Non-alcoholic fatty liver disease (NAFLD) is an expanding global health burden. NAFLD is an umbrella term that encompasses liver conditions ranging from steatosis to non-alcoholic steatohepatitis (NASH) and cirrhosis. While steatosis represents abnormal fat accumulation in the liver, NASH is steatosis accompanied by inflammation with or without fibrotic scarring (1). NASH can further progress to end-stage liver diseases such as cirrhosis and hepatocellular carcinoma (2). Though there have been considerable advances in recent years in elucidating the pathophysiological mechanisms involved in NASH, much of the underlying mechanisms remain elusive, which is hampering drug development (3, 4). Currently, no Food and Drug Administration (FDA)-approved drugs are available for this disease (5, 6) and therefore, there is an urgent need for effective treatment options to manage the complex pathogenesis of NASH.

We have previously demonstrated the relationship between lysosomal lipid accumulation in macrophages and hepatic inflammation during NASH (7, 8). Excessive accumulation of lipids in the lysosomes or changes in lysosomal pH leads to lysosomal dysfunction which subsequently disrupts the trafficking and sorting of lysosomal proteases causing their leakage into the circulation (9–12). In particular, the secreted lysosomal proteases, cathepsins, are known to participate in several inflammatory responses. For example, in bone disorders and cancer, cathepsins secretion is known to be a common part of the inflammatory response (13, 14).

CTSD is an aspartyl lysosomal protease synthesized in the rough endoplasmic reticulum from where it is sorted to lysosomes (15). As a result of excess lipids, extracellular CTSD secretion is elevated, where its increased activity in the plasma is known to be associated with metabolic inflammatory disorders such as NAFLD (16), atherosclerosis (17) and type 2 diabetes (18). We have established that inhibition of CTSD proteolytic activity using the generic aspartyl protease inhibitor pepstatin A ameliorated NASH (19). However, given the essential role of intracellular CTSD fraction in important cellular processes such as protein degradation and autophagy, its inhibition can have harmful effects if applied in humans (20, 21). Taking this into consideration, we have recently designed and tested a novel non-toxic inhibitor, CTD-002, that specifically targets the extracellular CTSD fraction. Our promising results showed that CTD-002 reduces hepatic steatosis and improves insulin sensitivity in Sprague-Dawley rats (22). While we successfully demonstrated the importance of extracellular CTSD activity in steatosis, the underlying mechanism of extracellular CTSD action compared to intracellular CTSD inhibition in NASH-associated hepatic inflammation was never investigated. Such evaluation is important in order to establish extracellular CTSD as an effective and less-toxic treatment for NASH.

By using specific small-molecule inhibitors of CTSD, we demonstrated that while intracellular CTSD is involved in essential processes such as mitochondrial oxidative phosphorylation and electron transport function, extracellular CTSD is mainly involved in pathways related to lipids and inflammation. In line, extracellular CTSD inhibition led to increased beneficial effects in NASH compared to intracellular CTSD inhibition, including reduced hepatic triglyceride levels, increased faecal bile acids and reduced systemic inflammation.

## Methods

### Primary Culture of Bone Marrow-Derived Macrophages (BMDMs)

BMDMs were isolated from the tibiae and femurs of wildtype C57BL/6 mice. Cells were cultured in RPMI-1640 (GIBCO Invitrogen, Breda, the Netherlands) with 10% heat-inactivated fetal calf serum (Bodinco B.V. Alkmaar, The Netherlands), penicillin (100 U/ml), streptomycin (100 μg/ml) and L-glutamine (2 mM) (all GIBCO Invitrogen, Breda, The Netherlands), supplemented with 20% L929-conditioned medium (LCM) for 8–9 days to generate BMDMs. After attachment, macrophages were seeded at 350,000 cells per well in 24-well plates and incubated for 24 hours with oxidized low-density lipoprotein (oxLDL) (25 μg/ml; Alfa Aesar, Wardhill, MA, USA). Subsequently, macrophages were treated with 100 µM CTD-002 or GA-12 (Aten Porus Lifesciences Pvt Ltd., India) or with vehicle control [0.1% dimethyl sulphoxide (DMSO)] for 4 hours. CTD-002 is a potent small-molecule active site inhibitor of CTSD designed based on the binding interactions of pepstatin A. Docking studies showed that CTD-002 interacts with polar side chain residues of ASP33 and GLY133 of ligand binding site of the human CTSD (PDB ID: 4od9).

### Mice, Diet and Intervention

*Ldlr^-/-^* mice, on a C57BL/6 background, were housed under standard conditions and given free access to food and water. Experiments were performed according to the Dutch regulation and approved by the Committee for Animal Welfare of Maastricht University (Project license number: AVD107002016743; working protocol: 2016-003-003). Female *Ldlr^-/-^* mice (8-14 weeks of age) were fed high-fat, high-cholesterol (HFC) diet (containing 21% milk butter, 0.2% cholesterol, 46% carbohydrates and 17% casein; SAFE, Augy, France) for ten weeks and were subsequently divided into three groups (n=17-20 per group; schematic overview in **Figure S1**). To evaluate the potential side effects of intracellular CTSD inhibition, a specific small-compound inhibitor, GA-12, was designed and used in this study. HFC-fed mice were subcutaneously injected with extracellular CTSD inhibitor (CTD-002; 50 mg/kg body weight) or intracellular CTSD inhibitor, GA-12 (50 mg/kg body weight) once every two days. Vehicle treatment (5% DMSO, 40% PEG400, 10% Ethanol, 45% Saline) served as control. *Ldlr^-/-^* mice fed a regular chow diet (9% fat, 67% carbohydrates and 24% protein) for 10 weeks were included as a control group for NASH disease phenotype (n=20). At the end of treatment, animals were euthanized by CO_2_ inhalation. Collection of blood and tissue specimens, RNA isolation and cDNA synthesis were performed as described previously (7, 19).

### Liver Lipid Analysis

Liver tissue was isolated and snap-frozen in liquid nitrogen and stored at -80°C. The biochemical determination of liver cholesterol and triglyceride levels were carried out as described previously (7). Briefly, 50 mg of frozen liver tissue was homogenized for 30 seconds at 5000 rpm in a closed tube with 1.0-mm glass beads and 1.0 ml SET buffer (Sucrose 250 mmol/l, EDTA 2 mmol/l and Tris 10 mmol/l). Complete cell destruction was done by 2 freeze-thaw cycles and 3 times passing through a 27-gauge syringe needle and a final freeze-thaw cycle. Protein content was measured using the BCA method Pierce, Rockford, IL). Liver cholesterol and triglyceride levels were quantified using cholesterol liquicolor kit [CHOD-PAP, Roche, Basel, Switzerland; triglycerides: GPOtrinder, TRO100, Sigma Aldrich, St. Louis, MO, USA)] following the manufacturer’s protocol on a Benchmark 550 micro-plate reader (Bio-Rad, Hercules, CA, USA).

### Liver Histology

Immunostainings were carried out on frozen liver sections (7µm) as described previously (23). Briefly, cryosections were fixated in dry acetone followed by blocking with H_2_O_2_ solution. Next, tissue sections were treated with avidin/biotin solution (Vector; SP2001) for 30 minutes followed by 1 hour incubation with primary antibody [infiltrated macrophages marker, Mac-1; MAB1124; clone M1/70; 1:500)], [F4/80;101201; Biolegio; (1:50)]. Sections later were incubated in secondary antibody (Rabbit anti-Rat IgG Biotin (6180-08), Southern Biotech, Birmingham, USA) which was detected using the Peroxidase Substrate kit AEC (Vector Laboratories, SK-4200, Peterborough, United Kingdom). Haematoxylin was used for nuclear counterstaining. Lipid and collagen content was assessed by using neutral lipid marker Oil Red O (ORO; O0625; Sigma-Aldrich) and Sirius red, (Direct red 80; 43664; Sigma-Aldrich) respectively according to standard protocol. Images were captured with a Nikon digital camera DMX1200 and ACT-1 v2.63 software (Nikon Instruments Europe, Amstelveen, The Netherlands).

### CTSD Activity Measurements

CTSD activity was measured using the CTSD activity assay kit (MBL International, Woburn, MA) as described previously (16). Briefly, cell medium was incubated with CTSD substrate and reaction buffer for 1 hour at 37°C. Samples were measured using a fluorescence plate reader with a 328-nm excitation filter and 460-nm emission filter and CTSD activity is expressed as relative fluorescence units.

### FACS Analysis

Fluorescence activated cell sorting (FACS) analysis was performed as described previously (19). Tail vein blood was collected from mice before the start of the treatment (T0) as well at the end of the treatment (T1). Using Trucount tubes (BD Biosciences, Breda, The Netherlands), staining was performed according to the manufacturer’s instructions, to detect the monocyte population (NK1.1-Ly6G-CD11b+; Ly6C). Briefly, heparinized blood samples were mixed and incubated for 10 minutes in the dark at room temperature (RT) with CD16/32 antibody (eBioscience, Halle-Zoersel, Belgium) to block Fc receptor. Samples were then gently vortexed with the appropriate antibodies (PE Mouse Anti-Mouse NK-1.1 (1:100); APC-Cy™7 Rat Anti-Mouse Ly-6G (1:100); Anti-Mouse Ly-6C-APC (1:10) (Miltenyi, Bergisch Gladbach, Germany) and incubated in the dark at RT for 20 minutes (24). All antibodies were diluted in FACS buffer (PBS, 0.1% BSA, 0.01% sodium azide). Finally, samples were mixed and incubated in the dark at RT for 15 minutes with an RBClysis solution (8.4g NH_4_Cl + 0.84g NAHCO_3_ in 1-liter H_2_O, pH 7.2-7.4). Sample stainings were quantified using BD FACSCanto II flow cytometer (BD Biosciences).

### Faecal Bile Acid Measurements

Faecal measurement was performed on faeces collected after 24 hours from individually caged mice. Total faecal bile acid levels were specifically determined as described previously (25).

### Label-Free LC-MS (Liquid Chromatography Mass Spectrometry) Based Proteomics

For proteomics experiments, liver homogenates were used (n=3 for each group of mice). The samples were prepared as described previously (26). Briefly, proteins were extracted, reduced with 20 mM dithiothreitol for 45 minutes and alkylated with 40 mM iodoacetamide for 45 minutes in the dark. Alkylation was terminated using 20 mM DDT which is followed by digestion with a mixture of LysC and trypsin at a ratio of 1:25 (enzyme:protein) at 37°C overnight. The digestion was stopped by formic acid. Peptide separation was performed on a Thermo Scientific (Dionex) Ultimate 3000 Rapid Separation UHPLC system equipped with a PepSep C18 analytical column (15 cm, ID 75 µm, 1,9 µm Reprosil, 120Å). Peptide samples were first desalted on an online installed C18 trapping column. After desalting peptides were separated on the analytical column with a 90-minute linear gradient from 5% to 35% Acetonitrile (ACN) with 0.1% FA at 300 nL/min flow rate. The UHPLC system was coupled to a Q Exactive HF mass spectrometer (Thermo Scientific). DDA settings were as follows. Full MS scan between 250 – 1,250 m/z at resolution of 120,000 followed by MS/MS scans of the top 15 most intense ions at a resolution of 15,000.

For protein identification and quantification, DDA spectra were analyzed with Proteome Discoverer version 2.2. The search engine Sequest was used with the Swiss-Prot mouse database (Mus musculus, TaxID 10090). The settings for the database search were similar as before (26). Proteins with a false- discovery rate ≤ 1% were considered for the analysis and were normalized to the total peptide amount. Statistical significance of changes observed in protein abundance was performed using ANOVA. The Benjamini– Hochberg method was used to correct P-values for multiple testing. Proteins were considered differently regulated if the fold change was ≥ 1.5 (log2 ≥0.58) and a p-value of ≤0.05. The differentially regulated proteins were then imported to the EnrichR tool and KEGG database (Verison 2019, mouse) was used to demonstrate the top 10 pathways of up or down-regulated proteins ranked by the combined score which is calculated by multiplying the unadjusted, rather than the adjusted, p-values with the z-scores (27).

### Statistical Analyses

Except for proteomics, rest of the data were analysed using GraphPad Prism software (GraphPad, San Diego, California, USA), version 6.0 for Windows. Experimental groups were compared using two-tailed student’s t-test or two-way ANOVA where appropriate or by one-way ANOVA followed by a Tukey post-hoc test. Data are expressed as mean ± SEM and were considered significant at p<0.05, in which *p<0.05; **p<0.01; or ***p<0.001 respectively.

## Results

### Cathepsin D Activity Is Reduced Using Low-Dose Extracellular CTSD Inhibitor

**Figure 1:**
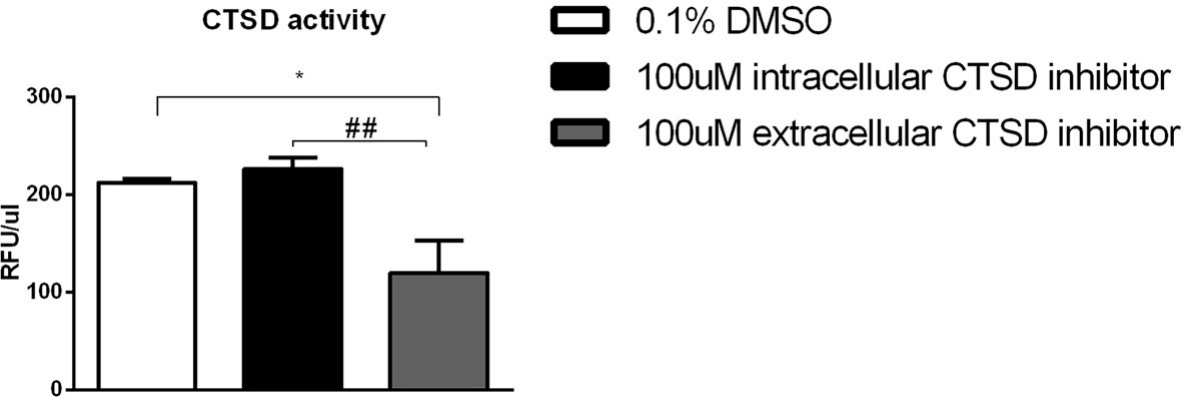
Efficacy of the cathepsin inhibitors: CTSD activity measurements from the supernatant of the *Wt* mouse BMDMs, exposed to 24 hours and subsequently treated with either vehicle control (0.1% DMSO) or 100µM CTSD inhibitors for 4 hours. Error bars represent ± SEM; n = 5 from two independent experiments. * represents p < 0.05 compared to DMSO-treated BMDMs. ^##^ represents p < 0.01 compared to intracellular CTSD inhibitor- treated BMDMs by means of one-way ANOVA.

To evaluate their potency and specificity, both the intracellular and extracellular CTSD small-compound inhibitors were tested using *in vitro* methods. CTSD activity was measured from the medium of bone marrow-derived macrophages (BMDMs) that were treated with either of the inhibitors. CTD-002 (i.e., the extracellular CTSD inhibitor) at a concentration of 100 µM significantly blocked CTSD activity when compared to DMSO (vehicle) as measured from the supernatant of BMDMs. In contrast, the intracellular CTSD inhibitor, GA-12, had no effect on CTSD activity in the supernatant, thus confirming the selective targeting of respective inhibitors (**Figure 1**).

### Extracellular CTSD Inhibition Led to Reduced Hepatic Lipid Levels Compared to Intracellular CTSD Inhibition

**Figure 2:**
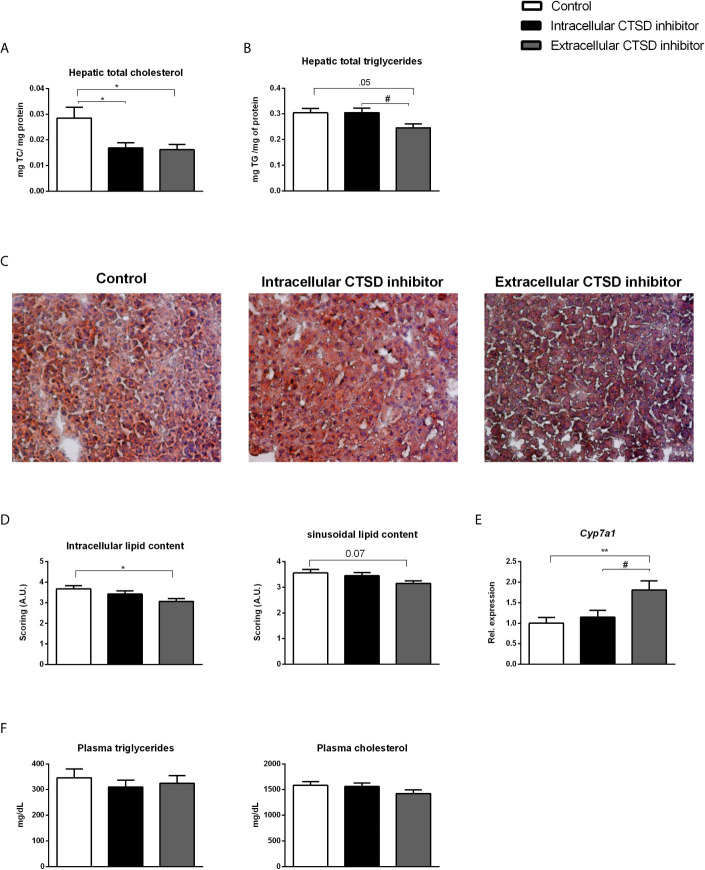
Hepatic and plasma lipids in hyperlipidaemic *Ldlr-/-* mice with or without CTSD inhibition: **(A, B)** Total hepatic cholesterol and triglyceride levels. **(C)** Representative pictures of oil red o staining on frozen liver sections (original magnification, 200x). **(D)** Scoring of hepatic lipid content of oil red o staining using arbitrary units (A.U.) **(E)** Hepatic gene expression analysis of *Cyp7a1.* **(F)** Plasma triglyceride and cholesterol measurements. Error bars represent ± SEM; n = 16-20 per each group; * represents p<0.05 and ** represents p < 0.01 compared to control mice. ^#^ represents p < 0.05 compared to intracellular CTSD inhibitor- treated mice by means of one-way ANOVA.

To test the effects of CTSD inhibition in NASH-associated dyslipidemia, *Ldlr-/-* mice on HFC diet were injected with either control or intra- or extracellular CTSD inhibitors for a period of 10 weeks. The small-compound CTSD inhibitors showed no visible signs of toxicity in mice. Food intake, body morphology and growth of all mice stayed normal throughout the 10-week experimental period. No abnormalities were detected in any of the animals at autopsy. To investigate the effects of CTSD inhibition on lipid metabolism, hepatic and plasma lipid levels were measured. After ten weeks of HFC diet, both plasma and liver cholesterol and triglycerides were significantly elevated in HFC-fed mice compared to mice on a chow diet, confirming the effect of HFC on hepatic and plasma lipid levels (**Figures S2A–D**). Further, treating HFC-fed mice with either the intracellular or the extracellular CTSD inhibitor decreased hepatic cholesterol levels compared to control mice (**Figure 2A**). However, only upon administration of the extracellular CTSD inhibitor, a reduction in hepatic triglyceride was observed (**Figure 2B**). To confirm the hepatic changes in lipid content at the histological level, we performed an oil red O staining which demonstrated elevated fat deposition in HFC mice compared to chow mice (**Figure S2C**). Moreover, in line with hepatic lipid measurements, extracellular CTSD-inhibitor treated mice showed significant reduction of intracellular and near significant reduction of sinusoidal lipid deposition compared to HFC mice (**Figures 2C, D**) suggesting a clear reduction in hepatic lipid content upon extracellular CTSD inhibition.

To further define the impact of extracellular CTSD inhibition on hepatic lipid metabolism, hepatic gene expression analysis using qPCR was performed. Compared to control and intracellular CTSD inhibitor-treated mice, inhibition of extracellular CTSD led to increased hepatic expression of cytochrome P450 7A1 (*Cyp7a1*), a rate-limiting enzyme in the breakdown of cholesterol to bile acids, suggesting increased cholesterol conversion only upon extracellular CTSD inhibition (**Figure 2E**). No pronounced effects in other lipid metabolism-related genes were observed (**Figure S4A**). However, plasma lipid levels remained similar among all experimental groups (**Figure 2F**). Altogether, these findings demonstrate that extracellular, rather than intracellular, CTSD inhibition decreases hepatic lipid accumulation in a mouse model for NASH.

### Extracellular CTSD Inhibition Led to Increased Faecal Bile Acid Levels Compared to Intracellular CTSD Inhibition

**Figure 3:**
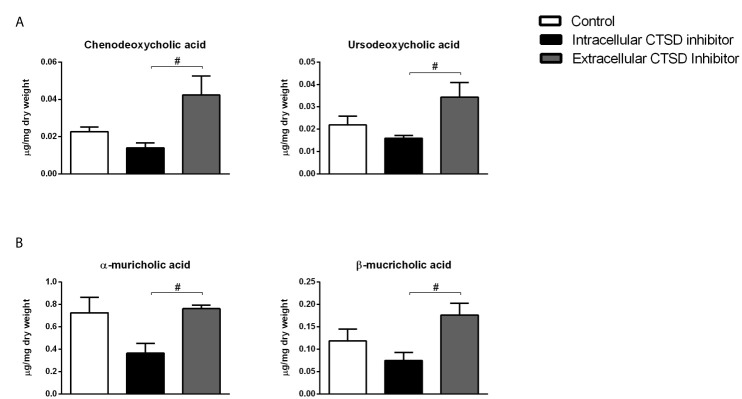
Increased faecal bile acid levels upon extracellular CTSD inhibition in *Ldlr-/-* mice: **(A)** Faecal measurements of primary bile acids **(B)** Secondary bile acids in the faeces. Error bars represent ± SEM. n = 4 per group. ^#^ represents p < 0.05 compared to intracellular CTSD inhibitor- treated mice as determined by one-way ANOVA.

To confirm the increased conversion of cholesterol into bile acid precursors, we opted to further investigate bile acid metabolism by assessing faecal bile acid levels. Faecal levels of primary bile acids, chenodeoxycholic acid (CDCA) and ursodeoxycholic acid (UDCA) were significantly enhanced in the extracellular inhibitor-treated group as compared to mice that received the intracellular inhibitor, indicating increased cholesterol breakdown in the former mice (**Figure 3A**). In line, secondary bile acids including α-muricholic acid and β-muricholic acid showed increased faecal levels in extracellular inhibitor-treated mice as compared to intracellular inhibitor-treated mice (**Figure 3B**). No differences in the faecal bile acid levels were observed between control and extracellular inhibitor-treated mice. Collectively, our data suggest that inhibition of extracellular CTSD activity in NASH mice results in increased disposal of cholesterol *via* increased conversion into bile acids leading to their subsequent excretion.

### Extracellular CTSD Inhibition Led to Reduced Systemic Inflammation Compared to Intracellular CTSD Inhibition

**Figure 4:**
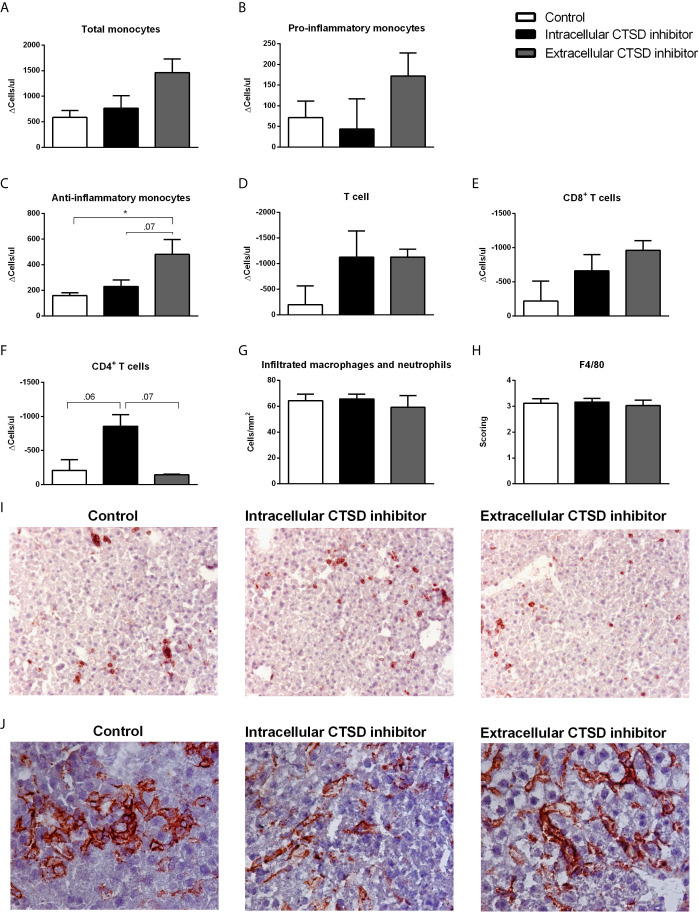
Reduction in inflammatory status upon extracellular CTSD inhibition in *Ldlr-/-* mice: **(A–F)** Total number of monocyte and T- cell population as measured by FACS. **(G)** Liver sections were stained for infiltrating macrophages and neutrophil cells (Mac-1). Positive cells were counted in six microscopical views. **(H)** Immunostaining of livers for F4/80. Positive cells were scored assessed and given a score in arbitrary units (A.U.) **(I)** Representative pictures of Mac-1 staining (original magnification, 200x) and **(J)** F4/80 (original magnification, 400x). Error bars represent SEM; n = 5 per each group for FACS and n = 3 per group for proteomics; * represents p < 0.05 compared to control mice as determined by one-way ANOVA.

Next, we examined the role of extracellular CTSD on inflammation at both hepatic and systemic levels. Firstly, inflammatory status was significantly elevated in HFC-fed mice compared to chow mice both at systemic and hepatic levels (**Figures S2E–G**). To explore the status of systemic inflammation upon extracellular CTSD inhibition, we analysed the profile of circulating monocytes using fluorescence-activated cell sorting (FACS). However, no statistical differences were observed in the number of total and pro-inflammatory monocytes (ΔT1-T0) between three different groups of HFC mice (**Figures 4A, B**). Further, indicating the decrease of systemic inflammation upon extracellular CTSD inhibition, extracellular inhibitor-treated mice showed a significant increase in the total number of anti-inflammatory monocytes (Ly6c-) compared to the control mice and intracellular CTSD inhibitor-treated mice (near significant; p=0.07) (**Figure 4C**). However, no significant changes in T-cell population were observed upon extracellular CTSD inhibition (**Figures 4D–F**). Together, these data demonstrate minor reduction in systemic inflammation upon extracellular CTSD inhibition. Next to systemic inflammation, the effects of extracellular CTSD inhibition on hepatic inflammation were assessed by hepatic immunostainings for the inflammatory cell markers Mac-1 and F4/80. The levels of infiltrated macrophages and F4/80 positive macrophage remained unaffected upon extracellular CTSD inhibition (**Figures 4G–J**). Lastly, as expected, the extent of hepatic fibrosis, an advanced NASH feature was no different in chow and experimental groups of HFC as demonstrated by Sirius red staining (**Figure S3**).

### Label-Free Quantitative Proteomics and Pathway Enrichment Analysis

To obtain a comprehensive understanding of the proteomic changes upon extracellular and intracellular CTSD inhibition in the liver, label-free quantitative proteomics was performed. After data processing, a total of 1905 proteins were identified. The number of significantly differentially regulated proteins were plotted using volcano plots (**Figure S5**). The complete list of significantly different regulated proteins (log2 ≥ 0.58) are mentioned in **Tables S1–S6**.

To further understand the functions of these differentially regulated proteins, enrichment analysis was performed to identify specific pathways. The top 10 KEGG (Kyoto encyclopaedia of genes and genomes) pathways that are most significantly enriched are displayed in **Table 1**. Compared to control and intracellular inhibitor-treated mice, extracellular inhibitor-treated mice showed enrichment of pathways related to lipid metabolism. Linoleic acid metabolism, steroid hormone biosynthesis, fatty acid biosynthesis and elongation were significantly enriched upon extracellular CTSD inhibition, suggesting improved lipid metabolism in these mice. Moreover, retinol metabolism, PPAR signalling, taurine/hypotaurine metabolism and caffeine metabolism were upregulated in extracellular inhibitor- treated mice (**Table 1A, B**). In addition to lipid-related pathways, ferroptosis, mineral absorption, quinone biosynthesis, ascorbate and aldarate metabolism, NAFLD and chemical carcinogenesis pathways were also enriched upon extracellular CTSD inhibition. Among the downregulated pathways in extracellular inhibitor-treated group, IL-17 pathway (proteins S100A8/A9), African trypanosomiasis, malaria (haemoglobin subunit beta 1), arginine and proline metabolism (creatinine kinase M-type) and glycosaminoglycan biosynthesis pathways (Xylosyltransferase 2) were in top 5 pathways compared to control (**Table 1C**). Interestingly, protein S100A8/A9, which is a ligand complex for Toll-like receptor 4 (TLR-4), was the most significantly downregulated protein suggesting that extracellular CTSD inhibition potentially reduces inflammation in a TLR-4 dependent manner. Moreover, pathways related to cardiac function (hypertrophic and dilated cardiomyopathy, cardiac muscle function), glycolysis, metabolic pathways (glycine, serine, threonine, thiamine, pyruvate, propanoate metabolism) and glucagon signalling were decreased in extracellular inhibitor-treated mice compared to intracellular CTSD inhibitor- treated mice (**Table 1D**).

**Table 1:** Differentially regulated proteins were imported to EnirchR tool and KEGG database was used for pathway enrichment analysis.

Name	P-value (p)	Combined Score
(A) KEGG database (Mouse) – extracellular inhibitor-treated mice/control	
Caffeine metabolism	0.005388	967.31
Linoleic acid metabolism	0.00001168	757.16
Biosynthesis of unsaturated fatty acids	0.0003734	548.11
Taurine and hypotaurine metabolism	0.009858	466.61
Ferroptosis	0.0005847	413.57
Mineral absorption	0.0007077	366.34
PPAR signalling pathway	0.002613	155.48
Steroid hormone biosynthesis	0.002860	146.24
Retinol metabolism	0.002988	141.96
Fatty acid elongation	0.02579	140.14
(B) KEGG database (Mouse) –extracellular inhibitor-treated mice/intracellular inhibitor-treated mice	
Steroid hormone biosynthesis	4,59E-12	824.33
Retinol metabolism	5,43E-09	535.98
Linoleic acid metabolism	1,59E-05	429.79
Chemical carcinogenesis	1,90E-07	407.05
Fatty acid biosynthesis	0.00006776	351.63
Peroxisome	6,11E-04	203.82
Ubiquinone and other terpenoid-quinone biosynthesis	0.001803	196.37
Non-alcoholic fatty liver disease (NAFLD)	2,59E-04	154.53
Ascorbate and aldarate metabolism	0.0005151	143.80
PPAR signalling pathway	0.00001057	138.25
(C) KEGG database (Mouse) –control/extracellular inhibitor-treated mice	
IL-17 signalling pathway	0.002357	166.22
African trypanosomiasis	0.03076	111.59
Malaria	0.03850	83.09
Arginine and proline metabolism	0.03927	80.93
Glycosaminoglycan biosynthesis	0.04158	75.00
Glutathione metabolism	0.05001	58.51
Metabolism of xenobiotics by cytochrome P450	0.05153	56.17
PPAR signalling pathway	0.06590	39.99
Protein digestion and absorption	0.06965	37.00
Chemical carcinogenesis	0.07263	34.87
(D) KEGG database (Mouse) –intracellular inhibitor-treated mice/extracellular inhibitor-treated mice	
Glycolysis/Gluconeogenesis	0.00004577	426.09
Hypertrophic cardiomyopathy (HCM)	0.00009653	307.16
Dilated cardiomyopathy (DCM)	0.0001105	289.22
Thiamine metabolism	0.01564	263.99
Cardiac muscle contraction	0.003005	141.82
Propanoate metabolism	0.03207	105.68
Glucagon signalling pathway	0.005077	98.65
Pyruvate metabolism	0.03917	81.20
Glycine, serine and threonine metabolism	0.04119	75.94
Adrenergic signalling in cardiomyocytes	0.01042	58.74

Top 10 enriched pathways ranked by combined score are listed below.

Most importantly, intracellular inhibitor-treated mice showed downregulation of proteins involved in mitochondrial oxidative phosphorylation and electron transport chain compared to control and extracellular inhibitor-treated mice (**Tables S1** and **S4**). According to KEGG pathways, intracellular inhibitor-treated mice showed enrichment of pathways belonging to muscle contraction and glycolysis compared to control (**Table S7**). Furthermore, proteins that are involved in hepatic stellate cell activation such as tropomyosin alpha-1 and beta chain, myosin regulatory light chain 2 and myosin 7 were upregulated. Taken together, these findings demonstrate that extracellular CTSD fraction regulates distinct pathways compared to intracellular CTSD in the context of NASH.

### Anti-Inflammatory Effects of Extracellular CTSD Inhibitor Are LPS-Dependent

**Figure 5:**
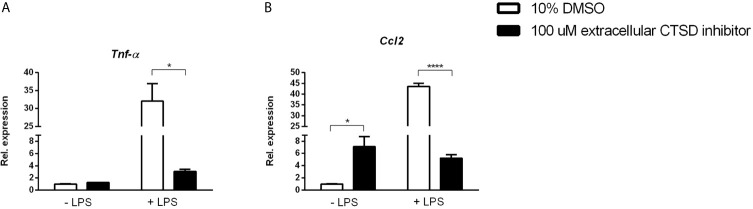
Anti-inflammatory effects of extracellular CTSD inhibitor are LPS- dependent: **(A, B)** Gene expression levels of *Tnfα*, and *Ccl2* from mouse *Wt* mouse BMDMs. Gene expression data are shown relative to DMSO-treated BMDMs with no LPS. Error bars represent ± SEM; n = 3 from an independent experiment. * represents p < 0.05 and **** represents p < 0.001 compared to DMSO-treated BMDMs as determined by two-way ANOVA.

To better understand the link between extracellular CTSD and TLR-4 mediated inflammation, we investigated whether lipopolysaccharide (LPS), a TLR4 agonist, influences the inflammatory effects of extracellular CTSD inhibitor. OxLDL-stimulated mouse bone-marrow derived macrophages were treated with extracellular CTSD inhibitor in the presence or absence of LPS. Only in the presence of LPS, extracellular CTSD inhibitor reduced gene expression levels of inflammatory cytokines, *Tnfα* and *Ccl2* (**Figures 5A, B**) suggesting that the anti-inflammatory effects of extracellular CTSD inhibitor are TLR-4 dependent.

## Discussion

Except for lifestyle interventions such as caloric restriction and exercise, there are currently no well-defined approved therapeutic strategies for NASH. As lifestyle changes are often insufficient, novel and safer pharmacologic treatments for NASH are urgently needed. While inhibition of CTSD activity was proven beneficial for NASH, the specific mechanisms by which extracellular and intracellular fractions of CTSD affect NASH were not explored. In the current study, we have identified that extracellular CTSD inhibition has more beneficial and less toxic effects in reducing metabolic inflammation over intracellular CTSD. These findings demonstrate the potential of extracellular CTSD to serve as a promising and safe target for the treatment of NASH.

Intracellular CTSD activity is accountable for cellular processes such as breakdown of intracellular proteins (28), cell signalling (29) and apoptosis (30). Therefore, CTSD knockout mice develop intestinal necrosis, destruction of lymphoid cells and myelin sheath atrophy leading to early death, suggesting CTSD as a key protein in physiology (21). In line, our proteomics data showed that inhibition of intracellular CTSD led to predominant downregulation of proteins specifically involved in mitochondrial oxidative phosphorylation and electron transport function namely Cox4i1, Cox6c, NADH dehydrogenase as well as CYP class of enzymes (Cyp2c29, Cyp3a41a, Cyp3a11, Cyp2c40, Cyp2c37, Cyp1a2, Cyp2e1) as found in 4/10 KEGG enriched pathways. It must be noted that downregulation of hepatic CYP enzymes might result in elevated plasma drug levels, adverse drug effects or drug-induced toxicity and inflammation (31) indicating unfavourable effects of intracellular CTSD inhibition. Besides executing its classical homeostatic activity inside the cells, CTSD also functions in the extracellular space both in physiological and pathological conditions (32). While controlled proteolytic activity of extracellular cathepsin D is evidently important for vital processes such as extracellular matrix (ECM) degradation during tissue repair, uncontrolled CTSD expression and activity is more commonly observed under pathological conditions (33, 34). Specifically, excessive extracellular CTSD activity has been implicated in metabolic inflammatory conditions (18, 22, 35, 36). In line with these compelling findings, we have demonstrated that extracellular CTSD activity is associated with NASH pathogenesis. Given evidence for the site-specific role of CTSD in pathophysiological processes, targeted blocking of its activity is critical to prevent any off-target side effects (37). To this end, selective effects of respective small-compound CTSD inhibitors obtained in our *in vitro* experiment strengthens the possibility that these inhibitors may have therapeutic value in treating NASH without causing disturbances of generalized lysosomal CTSD operations.

In our current study, we found pronounced effects of CTSD inhibition in the liver such as reduced lipid levels and alterations in metabolic pathways amongst others. This observation is in line with the concept that complex interplay between lipid acquisition and lipid disposal occurs in NASH (38). Lipids undergo constant recycling and relocation in the cell and lysosomes play an important role in this recycling as they regulate hepatic lipid catabolism and mobilization (39). In agreement, lysosomal dysfunction accompanied by aberrant cathepsin expression and activity is shown to be involved with the onset of NASH (16, 40–42). Similar to these observations, we have previously shown that CTSD plays a central role in lipid metabolism during NASH and that its inhibition reduces hepatic steatosis by biliary cholesterol elimination (19). The present study confirms the findings of this earlier report and provides insight for the specific role of extracellular CTSD fraction in lipid metabolism. In this study, pathways involved in hepatic lipid metabolism such as linoleic acid, fatty acid and steroid hormone biosynthesis have been found enriched in extracellular inhibitor-treated mice. The increased lipid biosynthesis upon extracellular CTSD inhibition likely reflects a compensatory response to the reduction in hepatic lipid levels and enhancement of cholesterol clearance. Further, amino acid (glycine, serine, and threonine) and pyruvate metabolism involving L-lactate dehydrogenase, enolase, and phosphoglycerate mutase proteins were downregulated suggesting reduced hepatic gluconeogenesis to compensate for the energy excess in the hyperlipidaemic mice. In addition to lipid-related pathways, proteomics analysis revealed enrichment of taurine and hypotaurine metabolism, retinol metabolism and PPAR signalling pathways upon extracellular CTSD inhibition. Taurine is an amino acid that is known to have diverse protective effects in liver diseases by suppressing diet-induced steatosis and inflammation among others (43). Noteworthy, retinoic acid and PPAR are known to synergically reverse liver fibrosis (44, 45). These findings further reinforce the potential benefits of extracellular CTSD inhibition in the context of liver diseases.

Furthermore, KEGG analysis revealed that IL-17 signalling pathway, drug and glutathione metabolism were downregulated upon extracellular CTSD inhibition. Relevantly, IL-17 signalling has been demonstrated to be involved in the pathogenesis of NASH. IL-17 pathway revealed the downregulation of S100A8/9 proteins. S100A8/9 proteins act as a ligand for Toll-like receptor 4 (TLR4) and are known to be released extracellularly, where they amplify inflammatory responses by inducing cytokine production such as IL-17 (46, 47). Moreover, TLR-4 activation is required for IL-17-mediated inflammation (48). Furthermore, both broad and selective cathepsin inhibitors are known to suppress IL-17 producing T helper cells during inflammatory process (49). Intriguingly, TLR4-dependent signalling is known to drive extracellular catabolism of modified lipoproteins by lysosomal proteases (50). These findings suggest that extracellular CTSD released upon hyperlipidaemic conditions might interact with S100A8/9 complex to activate the TLR4 signalling pathway which further exacerbates inflammation in a positive feedback mechanism.

It should however be taken into account that we could not detect profound effects of extracellular CTSD inhibition on other markers of hepatic inflammation. Moreover, while our data suggest an improvement in systemic inflammation, these data were retrieved from only a limited number of mice (n=5). More in-depth analysis including additional mice is therefore essential to confirm our findings. Further, 16 weeks of dietary intervention didn’t reveal significant differences in plasma inflammatory markers in low and HFC fed *Ldlr-/-* mice (51), suggesting that longer periods of diet are required to see such changes. As such the results of the current study are not sufficient to conclude that extracellular CTSD has major effects on reducing inflammation status. Therefore, it is currently unclear whether the dose of 50 mg/ kg of inhibitor and 10 weeks of HFC diet are sufficient to interfere with the inflammatory network. Future studies with higher and/or more frequent injections of CTSD inhibitor are warranted to answer these questions. Furthermore, it remains to be determined to what extent these potential mechanisms take place in humans.

Recently, we showed that extracellular CTSD inhibition led to reduced hepatic steatosis and insulin sensitivity in rats (22). However, it is noteworthy that the translational potential of these findings to humans is limited due to substantial differences in lipoprotein metabolism between humans and rats (52). For instance, hyperlipidaemic rats are known to display elevated plasma levels of high-density lipoprotein cholesterol (HDL-c) and reduced low-density lipoprotein cholesterol (LDL-c) levels, while humans show reversed plasma lipoprotein profiles, with higher levels of LDL-c and lower HDL-c. Relevantly, knocking out the LDL receptor in mice is known to block the uptake of LDL and results in a phenotype with excessive plasma LDL-c levels. Further, *Ldlr-/-* mice have the largest amount of the cholesterol in the form of IDL/LDL fraction (intermediate density lipoprotein/low-density lipoprotein) as seen in humans (53). Thus, *Ldlr-/-* mice possess the plasma lipoprotein profile as comparable to humans and serve as a better model to study the “human-like” profile (54). In agreement, *Ldlr -/-* mice have been used extensively as a research model to investigate pathways involved in human NASH (55–57).

The current study establishes a key role for extracellular CTSD in NASH-induced dyslipidaemia. Despite these significant findings, there are certain limitations to our study that include no advanced inflammatory data and absence of human data with CTSD inhibitors. Therefore, future studies using human models are warranted to expand our findings on inflammation and their validation in the clinical setting.

In conclusion, in contrast to the intracellular fraction of CTSD, extracellular CTSD inhibition has substantial benefits in restoration of lipid metabolism. Although the results of the current study should be interpreted with caution given the mild changes in inflammation, our data highlight that extracellular CTSD inhibition has favourable effects compared to intracellular inhibition and thus holds potential translational value as observed in a NASH mouse model with a ‘human-like’ lipoprotein profile.

## Data Availability Statement

The original contributions presented in the study are included in the article/**Supplementary Material**. Further inquiries can be directed to the corresponding author.

## Ethics Statement

The animal study was reviewed and approved by Committee for Animal Welfare of Maastricht University.

## Author Contributions

RS-S, TH, and YO conceived the study and designed the experiments. PK, SG, and AK designed and developed the inhibitors. TY, TH, AB, and JT contributed to sample collection, data collection, molecular experiments. MG assisted in animal sacrifice. RM performed proteomic studies. DL conducted faecal bile acid measurements. TY, TH, AB, RM, and BCP analysed the data. TY and TH wrote the manuscript. All authors helped in editing and revising the manuscript. RS-S obtained funding. All authors contributed to the article and approved the submitted version.

## Funding

This work was jointly supported by Avaliv Therapeutics and the Netherlands Organization for Scientific Research (NWO) VIDI (grant number: 016.126.327), ASPASIA (grant no. 015.008.043), and by Top consortia for Knowledge and Innovation’s (TKI; grant number: 40-41200-98-9306).

## Conflict of Interest

RS-S is on the advisory board and Aditya Kulkarni is a co-founder of Avaliv Therapeutics, which has filed intellectual property protecting the inhibitors. The terms of this arrangement have been reviewed and approved by Maastricht University in accordance with its policy on objectivity in research. PK, SG, and AK are employees of Aten Porus Lifesciences which carried out the chemistry related work for development of the inhibitors.

The remaining authors declare that the research was conducted in the absence of any commercial or financial relationships that could be construed as a potential conflict of interest.

## Abbreviations

BMDM, Bone-marrow derived macrophages; CDCA, chenodeoxycholic acid; CTSD, cathepsin D; DDA, Data-dependent acquisition; DDT, Dithiothreitol; ECM, extracellular matrix; FDA, Food & Drug Administration; HFC, High fat, high cholesterol diet; KEGG, Kyoto encyclopaedia of genes and genomes; LCM, L2-conditioned medium; LDLr-/-, Low-density lipoprotein receptor knockout; LFQ, Label-free quantification; MS, Mass spectroscopy; NAFLD, Non-alcoholic fatty liver disease; NASH, Non-alcoholic steatohepatitis; oxLDL, Oxidised low density-lipoprotein; TLR, Toll-like receptor; UDCA, Ursodeoxycholic acid; UHPLC, Ultra-high-pressure liquid chromatography.
