# Supplementary material for: Inhibition of Extracellular Cathepsin D Reduces Hepatic Lipid Accumulation and Leads to Mild Changes in Inflammationin NASH Mice
Source: Front Immunol. 2021 Jul 16;12:675535. doi: 10.3389/fimmu.2021.675535 (PMC8323051; doi:10.3389/fimmu.2021.675535)
Supplement: Supplementary file 1 [file DataSheet_1.docx]

**Supplementary data**

**Inhibition of extracellular cathepsin D reduces hepatic lipid accumulation and leads to mild changes in inflammation in NASH mice**

***Short title: Extracellular cathepsin D in NASH***

Tulasi Yadati^1^, Tom Houben^1^, Albert Bitorina^1^, Yvonne Oligschlaeger^1^, Marion J. Gijbels^2,3^, Ronny Mohren^4^, Dieter Lütjohann^5^, Princy Khurana^6^, Sandeep Goyal^6^, Aditya Kulkarni^6^, Jan Theys^7^, Berta Cillero-Pastor^4^ and Ronit Shiri-Sverdlov^1^

**Supplementary Figure 1**

**
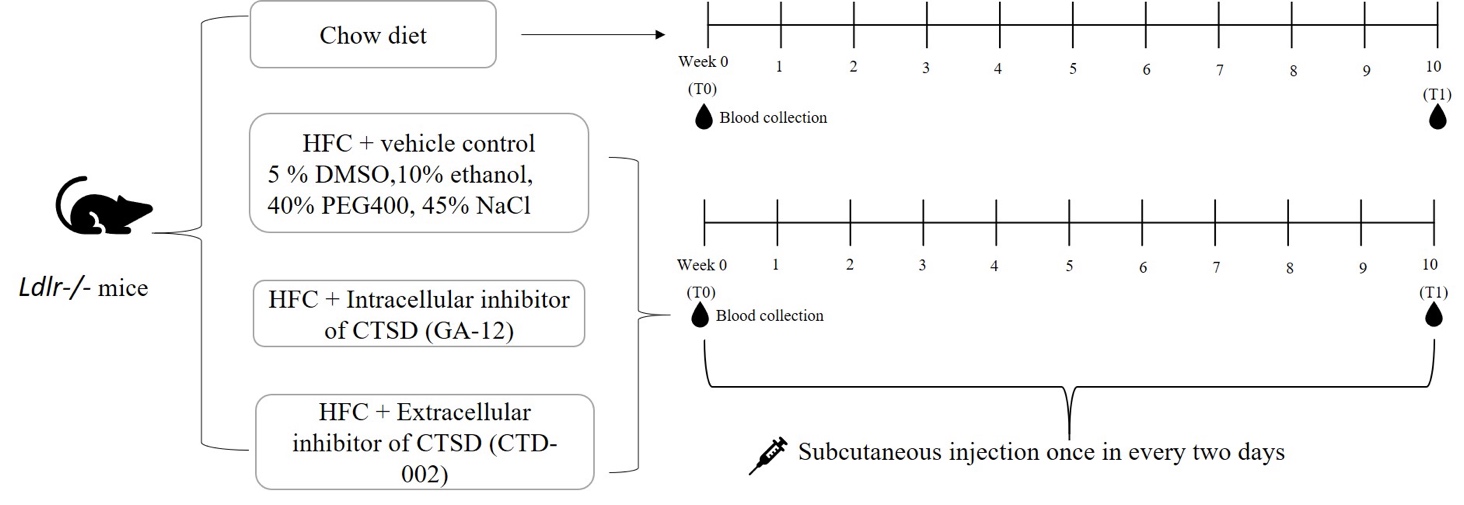
**

**Figure S1. Schematic representation of the study design and the timeline of the *in vivo* experiment**

Experimental timeline (Schematic drawing) of the *in vivo* experiment. *Ldlr^-/-^* mice fed a regular chow diet were included as a control group for NASH disease phenotype (n=20). HFC mice received vehicle or CTSD inhibitors subcutaneously once in every two days for 10 weeks. Sample sizes are n=17 for vehicle group, n=20 for intracellular inhibitor (GA-12)-treated group and n=17 for extracellular inhibitor (CTD-002)-treated group.

**Supplementary Figure 2**


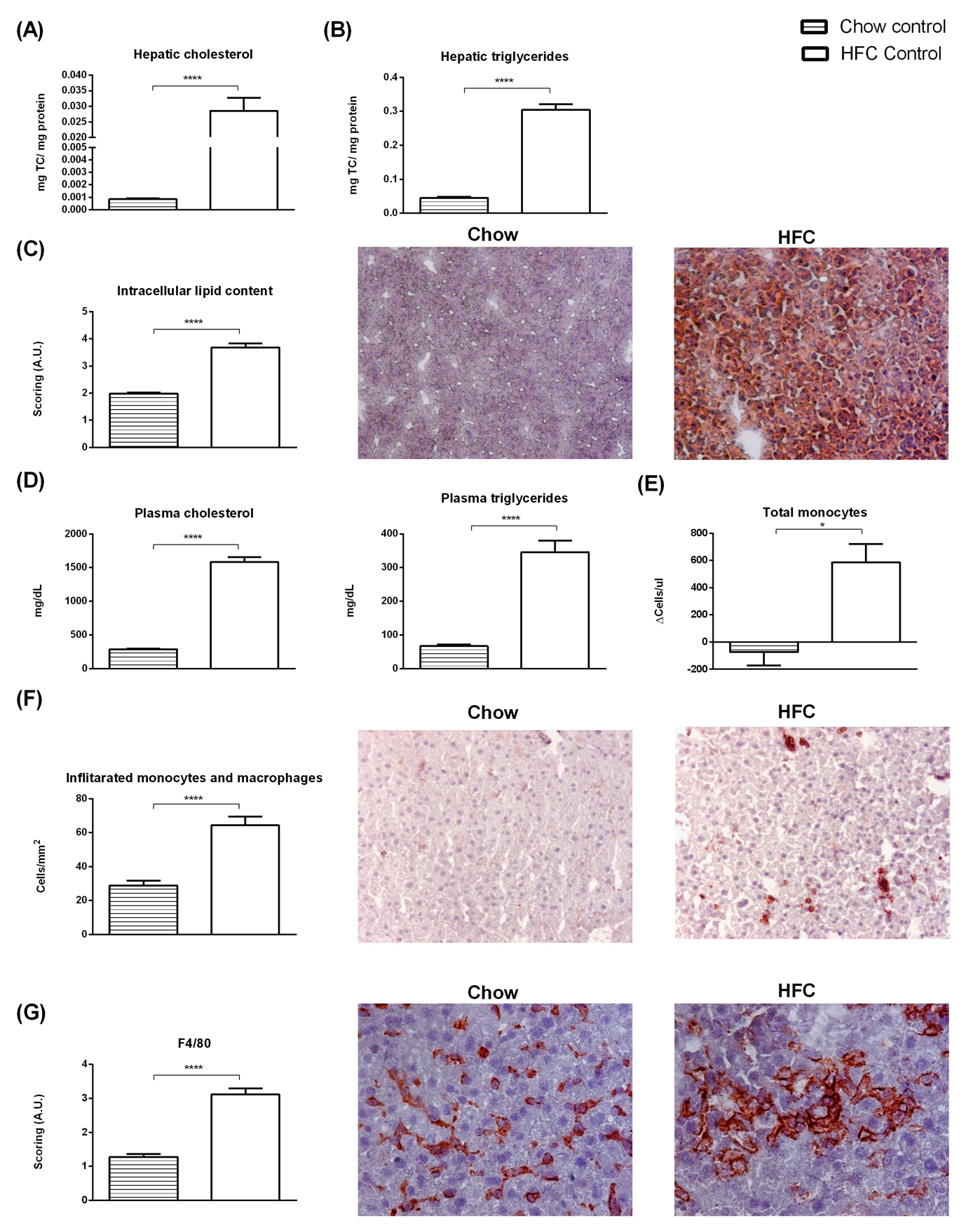


**Figure S2. HFC supplementation for 10 week induces NASH in *Ldlr-/-* mice**

**(A-B)** Feeding mice with HFC diet for 10 weeks lead to a significant increase in hepatic cholesterol and triglyceride levels compared to mice on chow diet. **(C)**  Scoring of hepatic lipid content (oil red o staining) using arbitrary units (A.U.). **(**Original magnification, 200x) **(D)** Plasma lipid levels between chow and HFC mice**. (E)** Total number of plasma monocytes as measured by FACS. **(F)** Liver sections were stained for infiltrating macrophages and neutrophils (Mac-1). Positive cells were counted in six microscopical views (original magnification, 200x) **(G)** Hepatic immunostaining for F4/80. Postive cells were evaluated and scored according to arbitrary units (original magnification, 400x)*.* Error bars represent ±SEM; n=17-20 animals; n=5 per each group for FACS; * represents p<0.05 **** represents p<0.001 compared to chow control mice as determined by two-tailed unpaired t test.

**Supplementary Figure 3**


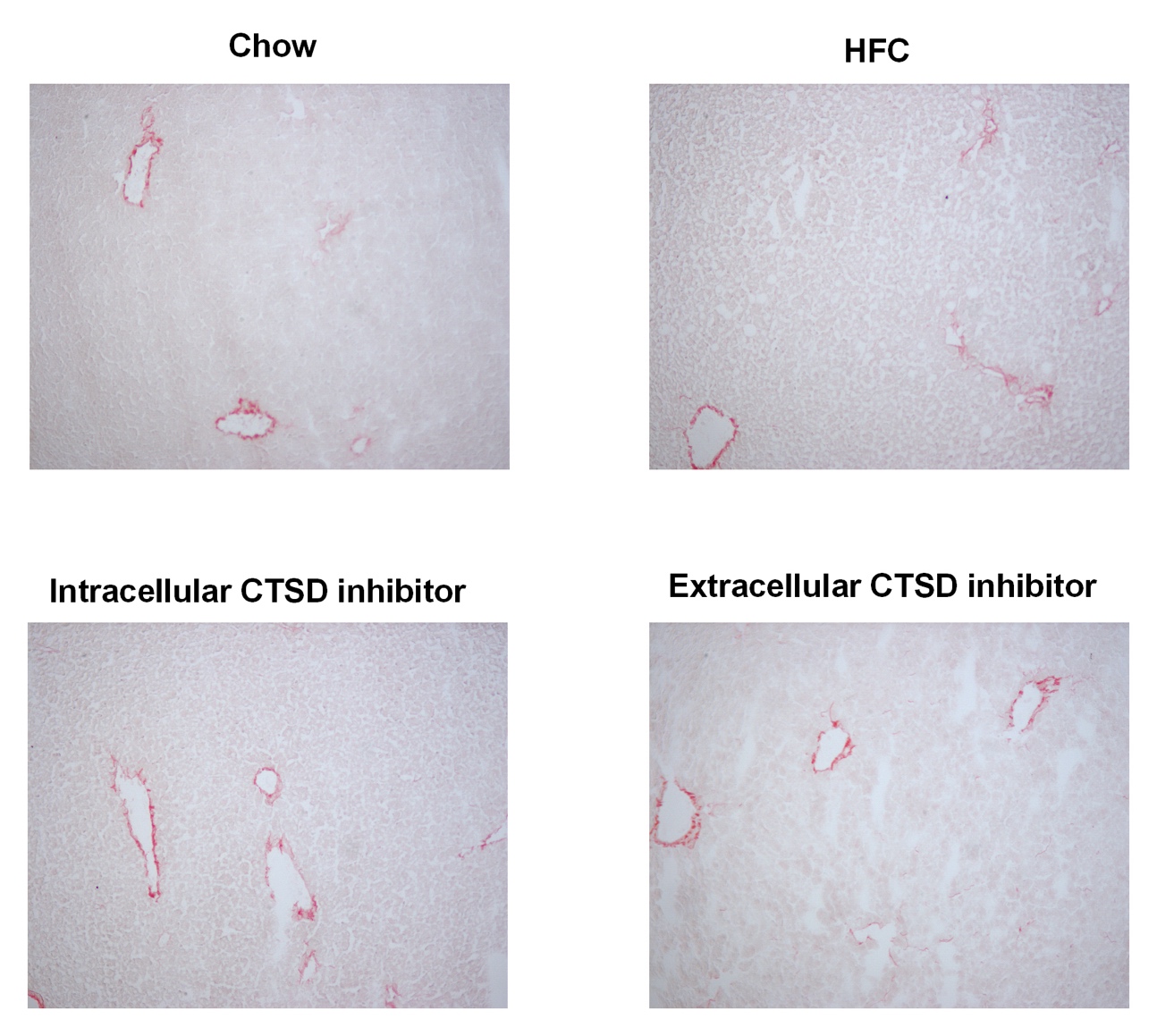


**Figure S3. No changes in hepatic fibrosis between different experimental groups of mice.**

Representative pictures of Sirius Red staining (original magnification, 100x)

**Supplementary Figure 4**

**
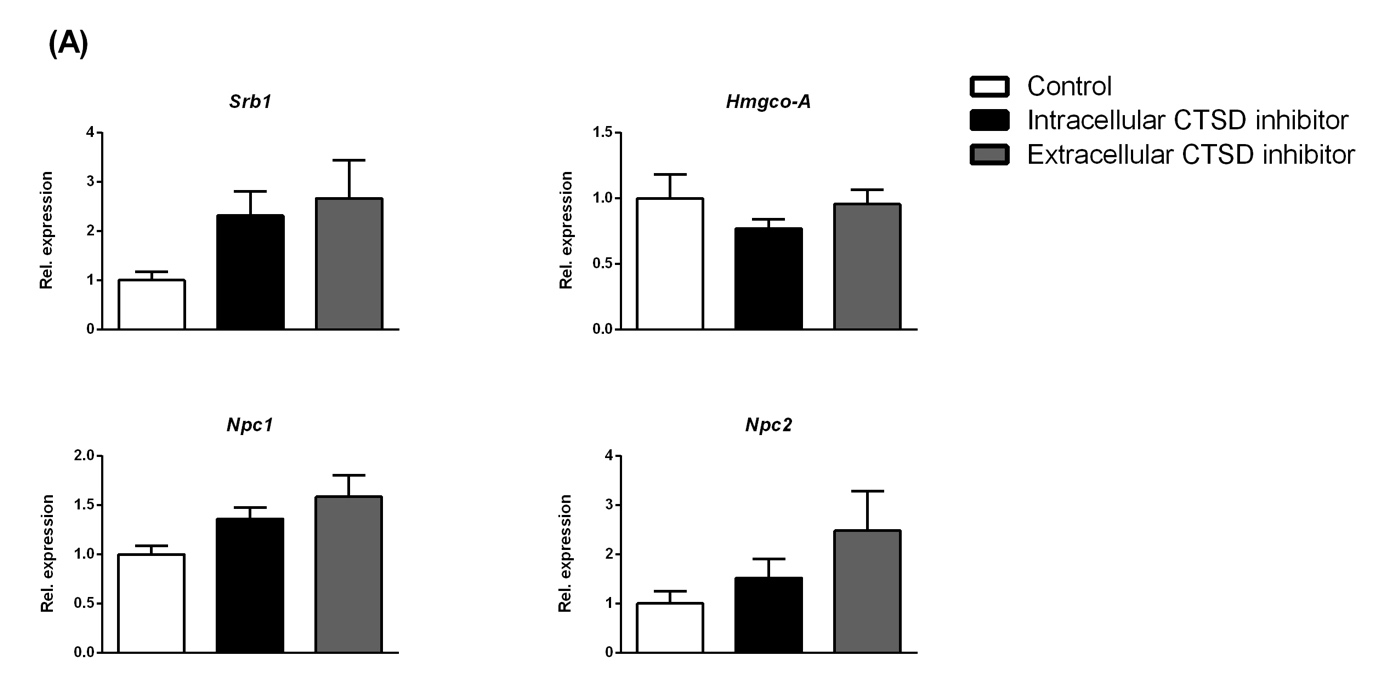
**

**Figure S4. Hepatic gene expression of lipid-related genes**

**(A)** Hepatic gene expression of lipid metabolism-related genes*.* Error bars represent ±SEM. n=16-20 per group.

**Supplementary Figure 5**

**(A)**

**(B)**

**(C)**

**Figure S5. Label-free quantitative LC-MS proteomics of different groups of mice.**

Volcano plots of protein changes observed in the livers of mice belonging to different groups. The −log10 (Benjamini–Hochberg corrected P value) is plotted against the log2 (fold change) for respective groups. **(A)** extracellular inhibitor-treated group vs control **(B)** intracellular inhibitor-treated group vs control and **(C)** extracellular vs intracellular inhibitor-treated groups.

**Table S1: Significantly upregulated proteins found in control mice compared to intracellular inhibitor-treated mice. Proteins were selected with a ratio of at least 1.5-fold change and p-value of ≤ 0.05. In the table, Protein accession number according to UniProt, protein names, gene ID, abundance ratio (log2 ≥0.58) are listed.**

|  | **Upregulated proteins in control mice compared to intracellular inhibitor-treated mice** |  | **Control/ intracellular inhibitor** |
| --- | --- | --- | --- |
| **Accession number** | **Description** | **Gene ID** | **Abundance ratio (log2)** |
| Q9D6Y9 | 1,4-alpha-glucan-branching enzyme | Gbe1 | 1,26 |
| Q9R092 | 17-beta-hydroxysteroid dehydrogenase type 6 | Hsd17b6 | 2,08 |
| P61922 | 4-aminobutyrate aminotransferase, mitochondrial | Abat | 1,4 |
| A0JNU3 | 60 kDa lysophospholipase | Aspg | 1,46 |
| Q9DCD0 | 6-phosphogluconate dehydrogenase, decarboxylating | Pgd | 1,78 |
| Q80XL6 | Acyl-CoA dehydrogenase family member 11 | Acad11 | 1,35 |
| Q99PL7 | Acyl-CoA desaturase | Scd3 | 3,31 |
| Q5XG73 | Acyl-CoA-binding domain-containing protein 5 | Acbd5 | 2,6 |
| P51881 | ADP/ATP translocase 2 | Slc25a5 | 1,75 |
| P47740 | Aldehyde dehydrogenase family 3 member A2 | Aldh3a2 | 1,79 |
| Q8BW75 | Amine oxidase [flavin-containing] B | Maob | 1,49 |
| Q91YI0 | Arginosuccinate lyase | Asl | 1,8 |
| P34927 | Asialoglycoprotein receptor 1 | Asgr1 | 1,79 |
| Q8BP47 | Asparagine--tRNA ligase, cytoplasmic | NARS1 | 1,79 |
| P04919 | Band 3 anion transport protein | Slc4a1 | 2,13 |
| P18572 | Basigin | Bsg | 1,61 |
| Q61335 | B-cell receptor-associated protein 31 | Bcap31 | 1,83 |
| Q9QXX4 | Calcium-binding mitochondrial carrier protein Aralar2 | Slc25a13 | 2,03 |
| Q61490 | CD166 antigen | Alcam | 1,77 |
| Q91WS0 | CDGSH iron-sulfur domain-containing protein 1 | Cisd1 | 2,14 |
| P60766 | Cell division control protein 42 homolog | Cdc42 | 2,37 |
| Q68FD5 | Clathrin heavy chain 1 | Cltc | 1,43 |
| Q9QZE5 | Coatomer subunit gamma-1 | Copg1 | 1,77 |
| Q9QZQ8 | Core histone macro-H2A.1 | H2afy | 1,89 |
| P50172 | Corticosteroid 11-beta-dehydrogenase isozyme 1 | Hsd11b1 | 1,88 |
| Q99L04 | Dehydrogenase/reductase SDR family member 1 | Dhrs1 | 2,59 |
| P50285 | Dimethylaniline monooxygenase [N-oxide-forming] 1 | Fmo1 | 2,72 |
| P97501 | Dimethylaniline monooxygenase [N-oxide-forming] 3 | Fmo3 | 2,38 |
| P97872 | Dimethylaniline monooxygenase [N-oxide-forming] 5 | Fmo5 | 2,57 |
| O54734 | Dolichyl-diphosphooligosaccharide--protein glycosyltransferase 48 kDa subunit | Ddost | 1,67 |
| Q91YQ5 | Dolichyl-diphosphooligosaccharide--protein glycosyltransferase subunit 1 | Rpn1 | 2,31 |
| P58252 | Elongation factor 2 | Eef2 | 1,66 |
| Q8BHI7 | Elongation of very long chain fatty acids protein 5 | Elovl5 | 3,2 |
| P19096 | Fatty acid synthase | Fasn | 2,24 |
| O08914 | Fatty-acid amide hydrolase 1 | Faah | 1,55 |
| P35576 | Glucose-6-phosphatase | G6pc | 2,12 |
| Q64516 | Glycerol kinase | Gk | 1,73 |
| Q9ET01 | Glycogen phosphorylase, liver form | Pygl | 1,51 |
| P01901 | H-2 class I histocompatibility antigen, K-B alpha chain | H2-K1 | 1,58 |
| P10922 | Histone H1. | H1-0 | 2,72 |
| P15864 | Histone H1.2 | H1-2 | 2,48 |
| P43274 | Histone H1.4 | H1-4 | 2,91 |
| P43276 | Histone H1.5 | H1-5 | 2,61 |
| P02301 | Histone H3.3C | H3f3c | 1,57 |
| P62806 | Histone H4 | H4c1 | 1,3 |
| Q8VCZ9 | Hydroxyproline dehydrogenase | Prodh2 | 2,82 |
| P01872 | Immunoglobulin heavy constant mu | Ighm | 1,43 |
| P70168 | Importin subunit beta-1 | Kpnb1 | 1,97 |
| Q9CXF0 | Kynureninase | Kynu | 1,67 |
| Q9WVM8 | Kynurenine/alpha-aminoadipate aminotransferase, mitochondrial | Aadat | 2,16 |
| Q922Q8 | Leucine-rich repeat-containing protein 59 | Lrrc59 | 2,76 |
| P58710 | L-gulonolactone oxidase | Gulo | 2,12 |
| P41216 | Long-chain-fatty-acid--CoA ligase 1 | Acsl1 | 1,68 |
| Q8JZR0 | Long-chain-fatty-acid--CoA ligase 5 | Acsl5 | 1,84 |
| O35114 | Lysosome membrane protein 2 | Scarb2 | 2,1 |
| Q99J39 | Malonyl-CoA decarboxylase, mitochondrial | Mlycd | 1,62 |
| O55022 | Membrane-associated progesterone receptor component 1 | Pgrmc1 | 1,58 |
| Q80UU9 | Membrane-associated progesterone receptor component 2 | Pgrmc2 | 2,33 |
| Q9DD20 | Methyltransferase-like protein 7B | Mettl7b | 2,33 |
| Q91VS7 | Microsomal glutathione S-transferase 1 | Mgst1 | 2,91 |
| Q922Q1 | Mitochondrial amidoxime reducing component 2 | Marc2 | 2 |
| Q9CW42 | Mitochondrial amidoxime-reducing component 1 | Marc1 | 1,79 |
| Q791V5 | Mitochondrial carrier homolog 2 | Mtch2 | 2,86 |
| Q9CZW5 | Mitochondrial import receptor subunit TOM70 | Tomm70 | 1,63 |
| Q9D023 | Mitochondrial pyruvate carrier 2 OS=Mus musculus OX=10090 GN=Mpc2 PE=1 SV=1 | Mpc2 | 1,93 |
| Q8VDD5 | Myosin-9 OS=Mus musculus | Myh9 | 1,71 |
| Q9ERS2 | NADH dehydrogenase [ubiquinone] 1 alpha subcomplex subunit 13 | Ndufa13 | 1,86 |
| O09111 | NADH dehydrogenase [ubiquinone] 1 beta subcomplex subunit 11, mitochondrial | Ndufb11 | 2,39 |
| Q9CQC7 | NADH dehydrogenase [ubiquinone] 1 beta subcomplex subunit 4 | Ndufb4 | 1,73 |
| Q9DC70 | NADH dehydrogenase [ubiquinone] iron-sulfur protein 7, mitochondrial | Ndufs7 | 2 |
| Q9DCN2 | NADH-cytochrome b5 reductase 3 | Cyb5r3 | 2,45 |
| Q9QXD1 | Peroxisomal acyl-coenzyme A oxidase 2 | Acox2 | 1,84 |
| P97372 | Proteasome activator complex subunit 2 | Psme2 | 2,13 |
| Q9CQS8 | Protein transport protein Sec61 subunit beta OS=Mus musculus OX=10090 GN=Sec61b PE=1 SV=3 | Sec61b | 3,18 |
| P35293 | Ras-related protein Rab-18 | Rab18 | 1,67 |
| P35278 | Ras-related protein Rab-5C | Rab5c | 2,09 |
| P51150 | Ras-related protein Rab-7a | Rab7a | 2,51 |
| Q9JM62 | Receptor expression-enhancing protein 6 | Reep6 | 1,49 |
| O88451 | Retinol dehydrogenase 7 | Rdh7 | 2,23 |
| Q9D5J6 | Sedoheptulokinase | Shpk | 1,89 |
| P52430 | Serum paraoxonase/arylesterase 1 | Pon1 | 1,45 |
| Q9JJL3 | Solute carrier organic anion transporter family member 1B2 | Slco1b2 | 3,72 |
| Q62465 | Synaptic vesicle membrane protein VAT-1 homolog | Vat1 | 2,13 |
| Q9D0R2 | Threonine--tRNA ligase 1, cytoplasmic | Tars1 | 2,12 |
| Q9QUI0 | Transforming protein RhoA | Rhoa | 2,27 |
| Q62186 | Translocon-associated protein subunit delta | Ssr4 | 1,97 |
| Q99KF1 | Transmembrane emp24 domain-containing protein 9 | Tmed9 | 2,41 |
| Q8JZU2 | Tricarboxylate transport protein, mitochondrial | Slc25a1 | 2,52 |
| P68373 | Tubulin alpha-1C chain | Tuba1c | 1,17 |
| P68372 | Tubulin beta-4B chain | Tubb4b | 1,21 |
| Q8BWQ1 | UDP-glucuronosyltransferase 2A3 | Ugt2a3 | 2,81 |
| P17717 | UDP-glucuronosyltransferase 2B17 | Ugt2b17 | 2,29 |
| Q8JZZ0 | UDP-glucuronosyltransferase 3A2 | Ugt3a2 | 2,78 |
| O35488 | Very long-chain acyl-CoA synthetase | Slc27a2 | 2,32 |
| O70503 | Very-long-chain 3-oxoacyl-CoA reductase | Hsd17b12 | 1,79 |
| Q9QY76 | Vesicle-associated membrane protein-associated protein B | Vapb | 2,09 |
| O08547 | Vesicle-trafficking protein SEC22b | Sec22b | 2,11 |
| P25444 | 40S ribosomal protein S2 | Rps2 | 1,32 |
| P97461 | 40S ribosomal protein S5 | Rps5 | 2,98 |
| Q6ZWV3 | 60S ribosomal protein L10 | Rpl10 | 1,24 |
| P19253 | 60S ribosomal protein L13a | Rpl13a | 1,5 |
| Q9CR57 | 60S ribosomal protein L14 | Rpl14 | 1,7 |
| Q9CZM2 | 60S ribosomal protein L15 | Rpl15 | 1,75 |
| P35980 | 60S ribosomal protein L18 | Rpl18 | 2,69 |
| Q8BP67 | 60S ribosomal protein L24 | Rpl24 | 1,71 |
| P41105 | 60S ribosomal protein L28 | Rpl28 | 1,74 |
| P47915 | 60S ribosomal protein L29 | Rpl29 | 1,55 |
| Q9D1R9 | 60S ribosomal protein L34 | Rpl34 | 2,36 |
| Q6ZWV7 | 60S ribosomal protein L35 | Rpl35 | 2,08 |
| P83882 | 60S ribosomal protein L36a | Rpl36a | 1,64 |
| Q9D8E6 | 60S ribosomal protein L4 | Rpl4 | 1,73 |
| P47911 | 60S ribosomal protein L6 | Rpl6 | 2,91 |
| P14148 | 60S ribosomal protein L7 | Rpl7 | 3,09 |
| P12970 | 60S ribosomal protein L7a | Rpl7a | 1,31 |
| Q9CQX2 | Cytochrome b5 type B | Cyb5b | 1,84 |
| P00405 | Cytochrome c oxidase subunit 2 | Mtco2 | 2,51 |
| P19783 | Cytochrome c oxidase subunit 4 isoform 1, mitochondrial | Cox4i1 | 1,96 |
| Q9CPQ1 | Cytochrome c oxidase subunit 6C | Cox6c | 2,19 |
| P20852 | Cytochrome P450 2A5 | Cyp2a5 | 2,78 |
| Q64458 | Cytochrome P450 2C29 | Cyp2c29 | 1,35 |
| P56654 | Cytochrome P450 2C37 | Cyp2c37 | 2,32 |
| P56657 | Cytochrome P450 2C40 | Cyp2c40 | 1,94 |
| P24456 | Cytochrome P450 2D10 | Cyp2d10 | 1,82 |
| Q8CIM7 | Cytochrome P450 2D26 | Cyp2d26 | 3,06 |
| Q05421 | Cytochrome P450 2E1 | Cyp2e1 | 2,68 |
| Q64459 | Cytochrome P450 3A11 | Cyp3a11 | 2,87 |
| Q9JMA7 | Cytochrome P450 3A41 | Cyp3a41a | 1,77 |
| Q9CQQ7 | ATP synthase F(0) complex subunit B1, mitochondrial | Atp5pb | 1,41 |
| P03930 | ATP synthase protein 8 | Mtatp8 | 1,97 |
| P56379 | ATP synthase subunit ATP5MPL, mitochondrial | Atp5mpl | 2,71 |
| Q9CPQ8 | ATP synthase subunit g, mitochondrial | Atp5mg | 1,63 |
| P55096 | ATP-binding cassette sub-family D member 3 | Abcd3 | 2,57 |

**Table S2: Significantly upregulated proteins found in control mice compared to extracellular inhibitor-treated mice. Proteins were selected with a ratio of at least 1.5-fold change and p-value of ≤ 0.05. In the table, Protein accession number according to UniProt, protein names, gene ID, abundance ratio (log2 ≥0.58) are listed.**

|  | **Upregulated proteins in response in control mice compared to extracellular inhibitor-treated mice** |  | **Control/ extracellular inhibitor** |
| --- | --- | --- | --- |
| **Accession number** | **Description** | **Gene Id** | **Abundance ratio (log2)** |
| Ca1 | Carbonic anhydrase 1 | Ca1 | 0,9 |
| Col14a1 | Collagen alpha-1(XIV) chain | Col14a1 | 0,82 |
| Ckm | Creatine kinase M-type | Ckm | 0,65 |
| Fabp3 | Fatty acid-binding protein, heart | Fabp3 | 0,69 |
| Gsta1 | Glutathione S-transferase A1 | Gsta1 | 0,66 |
| Hbb-b1 | Hemoglobin subunit beta-1 | Hbb-b1 | 0,65 |
| Ighv3-6 | Ig heavy chain V region 3-6 | Ighv3-6 | 1,28 |
| Nubpl | Iron-sulfur protein NUBPL | Nubpl | 0,59 |
| Mb | Myoglobin | Mb | 2,35 |
| Myl1 | Myosin light chain 1/3, skeletal muscle isoform | Myl1 | 1,04 |
| Prelp | Prolargin | Prelp | 0,74 |
| S100a8 | Protein S100-A8 | S100a8 | 0,83 |
| S100a9 | Protein S100-A9 | S100a9 | 0,65 |
| Spta1 | Spectrin alpha chain, erythrocytic 1 | Spta1 | 0,68 |
| Tnnc2 | Troponin C, skeletal muscle | Tnnc2 | 1,37 |
| Xylt2 | Xylosyltransferase 2 | Xylt2 | 0,91 |

**Table S3: Significantly upregulated proteins found in extracellular inhibitor-treated mice compared to control mice. Proteins were selected with a ratio of at least 1.5-fold change and p-value of ≤ 0.05. In the table, Protein accession number according to UniProt, protein names, gene ID, abundance ratio (log2 ≥0.58) are listed.**

|  | **Upregulated proteins in extracellular inhibitor-treated mice compared to control mice** |  | **Extracellular inhibitor/**  **control** |
| --- | --- | --- | --- |
| **Accession number** | **Description** | **Gene Id** | **Abundance ratio (log2)** |
| Q99PL7 | Acyl-CoA desaturase 3 | Scd3 | 0,6 |
| P07361 | Alpha-1-acid glycoprotein 2 | Orm2 | 1,12 |
| Q9DBE0 | Cysteine sulfinic acid decarboxylase | Csad | 0,8 |
| Q05816 | Fatty acid-binding protein 5 | Fabp5 | 0,71 |
| P01864 | Ig gamma-2A chain C region secreted form |  | 0,91 |
| P01655 | Ig kappa chain V-III region PC 7132 |  | 0,8 |
| Q8K2C9 | Very-long-chain (3R)-3-hydroxyacyl-CoA dehydratase 3 | Hacd3 | 0,86 |
| Q9R1Z8 | Vinexin | Sorbs3 | 0,68 |
| P18242 | Cathepsin D | Ctsd | 0,58 |
| O35403 | Amine sulfotransferase | Sult3a1 | 0,64 |
| P00186 | Cytochrome P450 1A2 | Cyp1a2 | 0,59 |
| O54749 | Cytochrome P450 2J5 | Cyp2j5 | 0,71 |
| Q64481 | Cytochrome P450 3A16 | Cyp3a16 | 1,12 |
| P04938 | Major urinary protein 11 | Mup11 | 0,66 |
| P11589 | Major urinary protein 2 | Mup2 | 0,61 |
| P09528 | Ferritin heavy chain | Fth1 | 0,85 |
| P29391 | Ferritin light chain 1 | Ftl1 | 0,9 |
| P46978 | Dolichyl-diphosphooligosaccharide--protein glycosyltransferase subunit STT3A | Stt3a | 0,72 |
| Q99KR7 | Peptidyl-prolyl cis-trans isomerase F, mitochondrial | Ppif | 0,71 |
| P54726 | UV excision repair protein RAD23 homolog A | Rad23a | 0,66 |

**Table S4: Significantly upregulated proteins found in extracellular inhibitor-treated mice compared to intracellular inhibitor-treated mice. Proteins were selected with a ratio of at least 1.5-fold change and p-value of ≤ 0.05. In the table, Protein accession number according to UniProt, protein names, gene ID, abundance ratio (log2 ≥0.58) are listed.**

|  | **Upregulated proteins in extracellular inhibitor-treated mice compared to Intracellular inhibitor-treated mice** |  | **Extracellular inhibitor/**  **intracellular**  **inhibitor** |
| --- | --- | --- | --- |
| **Accession number** | **Description** | **Gene ID** | **Abundance ratio (log2)** |
| P19096 | Fatty acid synthase | Fasn | 1,91 |
| Q9ET01 | Glycogen phosphorylase, liver form | Pygl | 1,66 |
| Q9DCN2 | NADH-cytochrome b5 reductase 3 | Cyb5r3 | 2,19 |
| Q791V5 | Mitochondrial carrier homolog 2 | Mtch2 | 2,42 |
| P35576 | Glucose-6-phosphatase | G6pc | 3,54 |
| Q91YI0 | Argininosuccinate lyase | Asl | 1,61 |
| P51881 | ADP/ATP translocase 2 | Slc25a5 | 1,66 |
| P41216 | Long-chain-fatty-acid--CoA ligase 1 | Acsl1 | 1,71 |
| Q9CZM2 | 60S ribosomal protein L15 | Rpl15 | 1,61 |
| P47740 | Aldehyde dehydrogenase family 3 member A2 | Aldh3a2 | 1,73 |
| P50172 | Corticosteroid 11-beta-dehydrogenase isozyme 1 | Hsd11b1 | 1,74 |
| Q9DD20 | Methyltransferase-like protein 7B | Mettl7b | 2,42 |
| P19783 | Cytochrome c oxidase subunit 4 isoform 1, mitochondrial | Cox4i1 | 1,79 |
| P01872 | Immunoglobulin heavy constant mu | Ighm | 1,72 |
| P58710 | L-gulonolactone oxidase | Gulo | 1,73 |
| Q922Q1 | Mitochondrial amidoxime reducing component 2 | Marc2 | 2 |
| O35488 | Very long-chain acyl-CoA synthetase | Slc27a2 | 1,7 |
| Q8JZR0 | Long-chain-fatty-acid--CoA ligase 5 | Acsl5 | 1,65 |
| Q9QXX4 | Calcium-binding mitochondrial carrier protein Aralar2 | Slc25a13 | 2,03 |
| P43274 | Histone H1.4 | H1-4 | 1,74 |
| O88451 | Retinol dehydrogenase 7 | Rdh7 | 1,79 |
| Q91WS0 | CDGSH iron-sulfur domain-containing protein 1 | Cisd1 | 1,83 |
| P00405 | Cytochrome c oxidase subunit 2 | Mtco2 | 2,35 |
| P97872 | Dimethylaniline monooxygenase [N-oxide-forming] 5 | Fmo5 | 2,29 |
| Q9CPQ1 | Cytochrome c oxidase subunit 6C | Cox6c | 2,35 |
| Q64516 | Glycerol kinase OS=Mus musculus OX=10090 GN=Gk PE=1 SV=2 | Gk | 1,78 |
| Q9DCD0 | 6-phosphogluconate dehydrogenase, decarboxylating | Pgd | 1,95 |
| Q9DCM2 | Glutathione S-transferase kappa 1 | Gstk1 | 1,7 |
| Q91YQ5 | Dolichyl-diphosphooligosaccharide--protein glycosyltransferase subunit 1 | Rpn1 | 1,86 |
| Q9CW42 | Mitochondrial amidoxime-reducing component 1 | Marc1 | 1,82 |
| Q9CXF0 | Kynureninase | Kynu | 1,73 |
| P03930 | ATP synthase protein 8 | Mtatp8 | 1,9 |
| Q8BP67 | 60S ribosomal protein L24 | Rpl24 | 1,58 |
| P35980 | 60S ribosomal protein L18 | Rpl18 | 2,06 |
| Q9D0R2 | Threonine--tRNA ligase 1, cytoplasmic | Tars1 | 2,1 |
| Q8CIM7 | Cytochrome P450 2D26 | Cyp2d26 | 2,6 |
| Q64458 | Cytochrome P450 2C29 | Cyp2c29 | 1,85 |
| Q99L04 | Dehydrogenase/reductase SDR family member 1 | Dhrs1 | 2,17 |
| P14148 | 60S ribosomal protein L7 | Rpl7 | 2,73 |
| P55096 | ATP-binding cassette sub-family D member 3 | Abcd3 | 2,27 |
| Q9QXD1 | Peroxisomal acyl-coenzyme A oxidase 2 | Acox2 | 2,03 |
| P47911 | 60S ribosomal protein L6 | Rpl6 | 1,86 |
| Q9D5J6 | Sedoheptulokinase | Shpk | 1,91 |
| P97372 | Proteasome activator complex subunit 2 | Psme2 | 1,69 |
| Q9QUI0 | Transforming protein RhoA | Rhoa | 2,34 |
| P50285 | Dimethylaniline monooxygenase [N-oxide-forming] 1 | Fmo1 | 2,07 |
| Q91X44 | Glucokinase regulatory protein | Gckr | 1,67 |
| P24456 | Cytochrome P450 2D10 | Cyp2d10 | 2,15 |
| Q9D1R9 | 60S ribosomal protein L34 | Rpl34 | 2,3 |
| P34927 | Asialoglycoprotein receptor 1 | Asgr1 | 1,61 |
| Q922Q8 | Leucine-rich repeat-containing protein 59 | Lrrc59 | 2,25 |
| Q9CZW5 | Mitochondrial import receptor subunit TOM70 | Tomm70 | 1,59 |
| P97461 | 40S ribosomal protein S5 | Rps5 | 2,85 |
| P15864 | Histone H1.2 | H1-2 | 1,69 |
| P10922 | Histone H1.0 | H1-0 | 1,83 |
| P17717 | UDP-glucuronosyltransferase 2B17 | Ugt2b17 | 2,28 |
| Q9CQC7 | NADH dehydrogenase [ubiquinone] 1 beta subcomplex subunit 4 | Ndufb4 | 1,67 |
| Q61335 | B-cell receptor-associated protein 31 | Bcap31 | 1,58 |
| Q99J39 | Malonyl-CoA decarboxylase, mitochondrial | Mlycd | 1,58 |
| Q9WVM8 | Kynurenine/alpha-aminoadipate aminotransferase, mitochondrial | Aadat | 2,02 |
| P60766 | Cell division control protein 42 homolog | Cdc42 | 2,38 |
| Q8BWQ1 | UDP-glucuronosyltransferase 2A3 | Ugt2a3 | 2,75 |
| Q62465 | Synaptic vesicle membrane protein VAT-1 homolog | Vat1 | 1,93 |
| P56654 | Cytochrome P450 2C37 | Cyp2c37 | 1,78 |
| P97501 | Dimethylaniline monooxygenase [N-oxide-forming] 3 | Fmo3 | 1,98 |
| P56135 | ATP synthase subunit f, mitochondrial | Atp5mf | 1,95 |
| Q9CQX2 | Cytochrome b5 type B | Cyb5b | 2,01 |
| O35114 | Lysosome membrane protein 2 | Scarb2 | 2,12 |
| Q8R1I1 | Cytochrome b-c1 complex subunit 9 | Uqcr10 | 1,77 |
| A2AKK5 | Acyl-coenzyme A amino acid N-acyltransferase 1 | Acnat1 | 1,59 |
| Q9DC70 | NADH dehydrogenase [ubiquinone] iron-sulfur protein 7, mitochondrial | Ndufs7 | 1,95 |
| O08547 | Vesicle-trafficking protein SEC22b | Sec22b | 1,73 |
| P61027 | Ras-related protein Rab-10 | Rab10 | 1,56 |
| Q62186 | Translocon-associated protein subunit delta | Ssr4 | 1,58 |
| P56657 | Cytochrome P450 2C40 | Cyp2c40 | 1,98 |
| Q9QY76 | Vesicle-associated membrane protein-associated protein B | Vapb | 1,79 |
| P18572 | Basigin | Bsg | 1,82 |
| Q64514 | Tripeptidyl-peptidase 2 | Tpp2 | 1,71 |
| Q8JZZ0 | UDP-glucuronosyltransferase 3A2 | Ugt3a2 | 1,88 |
| Q9D023 | Mitochondrial pyruvate carrier 2 | Mpc2 | 1,76 |
| Q8JZU2 | Tricarboxylate transport protein, mitochondrial | Slc25a1 | 2,32 |
| P00186 | Cytochrome P450 1A2 | Cyp1a2 | 2,39 |
| Q8BP47 | Asparagine--tRNA ligase, cytoplasmic | NARS1 | 1,72 |
| Q64459 | Cytochrome P450 3A11 | Cyp3a11 | 2 |
| Q5XG73 | Acyl-CoA-binding domain-containing protein 5 | Acbd5 | 2,45 |
| B1AR13 | CDGSH iron-sulfur domain-containing protein 3, mitochondrial | Cisd3 | 1,85 |
| O70503 | Very-long-chain 3-oxoacyl-CoA reductase | Hsd17b12 | 1,64 |
| Q9JMA7 | Cytochrome P450 3A41 | Cyp3a41a | 2,87 |
| O09111 | NADH dehydrogenase [ubiquinone] 1 beta subcomplex subunit 11, mitochondrial | Ndufb11 | 2,48 |
| Q8BHI7 | Elongation of very long chain fatty acids protein 5 | Elovl5 | 2,55 |
| Q8VCZ9 | Hydroxyproline dehydrogenase | Prodh2 | 2,91 |
| P51150 | Ras-related protein Rab-7a | Rab7a | 2,26 |
| P04919 | Band 3 anion transport protein | Slc4a1 | 1,67 |
| P35278 | Ras-related protein Rab-5C | Rab5c | 1,92 |
| Q9CR61 | NADH dehydrogenase [ubiquinone] 1 beta subcomplex subunit 7 | Ndufb7 | 1,81 |
| Q9R092 | 17-beta-hydroxysteroid dehydrogenase type 6 | Hsd17b6 | 1,82 |
| P56379 | ATP synthase subunit ATP5MPL, mitochondrial | Atp5mpl | 3,36 |
| Q925I1 | ATPase family AAA domain-containing protein 3 | Atad3 | 1,63 |
| Q80UU9 | Membrane-associated progesterone receptor component 2 | Pgrmc2 | 2,13 |
| Q99PL7 | Acyl-CoA desaturase | Scd3 | 2,8 |
| Q9CQS8 | Protein transport protein Sec61 subunit beta | Sec61b | 3,03 |
| Q9R1J0 | Sterol-4-alpha-carboxylate 3-dehydrogenase, decarboxylating | Nsdhl | 1,78 |
| Q99KF1 | Transmembrane emp24 domain-containing protein 9 | Tmed9 | 2,27 |
| Q9R1P3 | Proteasome subunit beta type-2 | Psmb2 | 1,72 |
| P15392 | Cytochrome P450 2A4 | Cyp2a4 | 2,21 |
| Q9CRC0 | Vitamin K epoxide reductase complex subunit 1 | Vkorc1 | 1,88 |
| O09117 | Synaptophysin-like protein 1 | Sypl1 | 1,79 |
| P20852 | Cytochrome P450 2A5 | Cyp2a5 | 2,92 |
| P43276 | Histone H1.5 | H1-5 | 1,57 |
| Q9CQW9 | Interferon-induced transmembrane protein 3 | Ifitm3 | 1,79 |
| Q9D2R0 | Acetoacetyl-CoA synthetase | Aacs | 2,02 |
| Q9CQC6 | Basic leucine zipper and W2 domain-containing protein 1 | Bzw1 | 1,68 |
| Q9JJL3 | Solute carrier organic anion transporter family member 1B2 | Slco1b2 | 3,87 |
| Q64669 | NAD(P)H dehydrogenase [quinone] | Nqo1 | 1,83 |
| Q76M72 | Solute carrier family 22 member 27 | Slc22a27 | 2,31 |
| Q61490 | CD166 antigen | Alcam | 1,65 |
| Q05421 | Cytochrome P450 2E1 | Cyp2e1 | 2,07 |
| P20852 | Cytochrome P450 2A5 | Cyp2a5 | 2,92 |
| P15392 | Cytochrome P450 2A4 | Cyp2a4 | 2,21 |
| Q9JMA7 | Cytochrome P450 3A41 | Cyp3a41a | 2,87 |
| Q64459 | Cytochrome P450 3A11 | Cyp3a11 | 2 |
| P00186 | Cytochrome P450 1A2 | Cyp1a2 | 2,39 |
| P56657 | Cytochrome P450 2C40 | Cyp2c40 | 1,98 |
| Q9CQX2 | Cytochrome b5 type B | Cyb5b | 2,01 |
| P56654 | Cytochrome P450 2C37 | Cyp2c37 | 1,78 |
| Q64458 | Cytochrome P450 2C29 | Cyp2c29 | 1,85 |
| Q8CIM7 | Cytochrome P450 2D26 | Cyp2d26 | 2,6 |
| Q9DCN2 | NADH-cytochrome b5 reductase 3 | Cyb5r3 | 2,19 |

**Table S5: Significantly upregulated proteins found in intracellular inhibitor-treated mice compared to control mice. Proteins were selected with a ratio of at least 1.5-fold change and p-value of ≤ 0.05. In the table, Protein accession number according to UniProt, protein names, gene ID, abundance ratio (log2 ≥0.58) are listed.**

|  | **Upregulated proteins in intracellular inhibitor-treated mice compared to control mice** |  | **Intracellular inhibitor/**  **control** |
| --- | --- | --- | --- |
| **Accession number** | **Description** | **Gene ID** | **Abundance ratio (log2)** |
| Q9R0Y5 | Adenylate kinase isoenzyme 1 | Ak1 | 4,1 |
| P21550 | Beta-enolase | Eno3 | 5,97 |
| O54724 | Caveolae-associated protein 1 | Cavin1 | 2,37 |
| P07310 | Creatine kinase M-type | Ckm | 4,05 |
| Q6P8J7 | Creatine kinase S-type, mitochondrial | Ckmt2 | 3,2 |
| P31001 | Desmin | Des | 2,42 |
| P11404 | Fatty acid-binding protein, heart | Fabp3 | 3,04 |
| P97447 | Four and a half LIM domains protein 1 | Fhl1 | 3,4 |
| P14602 | Heat shock protein beta-1 | Hspb1 | 1,98 |
| Q5EBG6 | Heat shock protein beta-6 | Hspb6 | 3,79 |
| Q9JKS4 | LIM domain-binding protein 3 | Ldb3 | 5,75 |
| P16125 | L-lactate dehydrogenase B chain | Ldhb | 2,11 |
| P04247 | Myoglobin OS=Mus musculus | Mb | 6,52 |
| Q62234 | Myomesin-1 OS=Mus musculus | Myom1 | 3,59 |
| P05977 | Myosin light chain 1/3, skeletal muscle isoform | Myl1 | 3,64 |
| P51667 | Myosin regulatory light chain 2, ventricular/cardiac muscle isoform | Myl2 | 4,68 |
| Q5SX40 | Myosin-1 OS=Mus musculus | Myh1 | 2,31 |
| Q5SX39 | Myosin-4 OS=Mus musculus | Myh4 | 1,81 |
| Q91Z83 | Myosin-7 OS=Mus musculus | Myh7 | 1,85 |
| Q9JK37 | Myozenin-1 | Myoz1 | 2,54 |
| Q9JJW5 | Myozenin-2 | Myoz2 | 1,95 |
| P00688 | Pancreatic alpha-amylase | Amy2 | 2,89 |
| O70250 | Phosphoglycerate mutase 2 | Pgam2 | 4,87 |
| Q9R1C7 | Pre-mRNA-processing factor 40 homolog A | Prpf40a | 2,27 |
| P58771 | Tropomyosin alpha-1 chain | Tpm1 | 3,35 |
| P58774 | Tropomyosin beta chain | Tpm2 | 4,93 |
| P20801 | Troponin C, skeletal muscle | Tnnc2 | 4,08 |
| P13412 | Troponin I, fast skeletal muscle | Tnni2 | 2,25 |
| Q9QZ47 | Troponin T, fast skeletal muscle | Tnnt3 | 2,53 |
| Q11136 | Xaa-Pro dipeptidase | Pepd | 3,81 |

**Table S6: Significantly upregulated proteins found in intracellular inhibitor-treated mice compared to extracellular inhibitor-treated mice. Proteins were selected with a ratio of at least 1.5-fold change and p-value of ≤ 0.05. In the table, Protein accession number according to UniProt, protein names, gene ID, abundance ratio (log2 ≥0.58) are listed.**

|  | **Upregulated proteins in intracellular inhibitor-treated mice compared to extracellular inhibitor-treated mice** |  | **Intracellular inhibitor/ extracellular inhibitor** |
| --- | --- | --- | --- |
| **Accession number** | **Description** | **Gene ID** | **Abundance ratio (log2)** |
| Q5SX40 | Myosin-1 | Myh1 | 2,54 |
| P07310 | Creatine kinase M-type | Ckm | 5,61 |
| P58771 | Tropomyosin alpha-1 chain | Tpm1 | 3,31 |
| P05977 | Myosin light chain 1/3, skeletal muscle isoform | Myl1 | 3,07 |
| P58774 | Tropomyosin beta chain | Tpm2 | 5,16 |
| Q9QZ47 | Troponin T, fast skeletal muscle | Tnnt3 | 3,48 |
| P21550 | Beta-enolase | Eno3 | 4,56 |
| P31001 | Desmin | Des | 2,71 |
| P13412 | Troponin I, fast skeletal muscle | Tnni2 | 2,42 |
| P97447 | Four and a half LIM domains protein 1 | Fhl1 | 3,47 |
| P11404 | Fatty acid-binding protein, heart | Fabp3 | 6,52 |
| Q9R0Y5 | Adenylate kinase isoenzyme 1 | Ak1 | 3,89 |
| O70250 | Phosphoglycerate mutase 2 | Pgam2 | 4,04 |
| Q62234 | Myomesin-1 | Myom1 | 3,85 |
| Q5SX39 | Myosin-4 | Myh4 | 2,08 |
| P16125 | L-lactate dehydrogenase B chain | Ldhb | 2,03 |
| Q9JK37 | Myozenin-1 | Myoz1 | 2,45 |
| P14602 | Heat shock protein beta-1 | Hspb1 | 2,14 |
| O54724 | Caveolae-associated protein 1 | Cavin1 | 2,33 |
| Q9JJW5 | Myozenin-2 | Myoz2 | 2,11 |
| Q91WK1 | SPRY domain-containing protein 4 | Spryd4 | 1,62 |
| P14602 | Heat shock protein beta-1 | Hspb1 | 2,14 |

**Table S7: Top 10 enriched pathways upon intracellular CTSD inhibition**

KEGG database (Mouse) – Intracellular inhibitor-treated mice/ control

| Name | P-value (p) | Combined Score |
| --- | --- | --- |
| Hypertrophic cardiomyopathy (HCM) | 1.711e-7 | 603.92 |
| Dilated cardiomyopathy (DCM) | 2.150e-7 | 568.61 |
| Cardiac muscle contraction | 0.000005430 | 414.48 |
| Glycolysis / Gluconeogenesis | 0.0001367 | 265.60 |
| Adrenergic signalling in cardiomyocytes | 0.00006793 | 172.92 |
| Thiamine metabolism | 0.02227 | 169.08 |
| Arginine and proline metabolism | 0.002548 | 159.27 |
| Tight junction | 0.001967 | 74.63 |
| Propanoate metabolism | 0.04550 | 66.45 |
| Glucagon signalling pathway | 0.01021 | 59.93 |

KEGG database (Mouse) –control/ intracellular inhibitor-treated mice

| Name | P-value (p) | Combined Score |
| --- | --- | --- |
| Steroid hormone biosynthesis | 6.332e-13 | 573.81 |
| Ribosome | 1.294e-15 | 519.41 |
| Fatty acid biosynthesis | 0.00009699 | 300.03 |
| Retinol metabolism | 8.955e-9 | 277.69 |
| Chemical carcinogenesis | 1.195e-8 | 264.64 |
| Linoleic acid metabolism | 9.232e-7 | 252.64 |
| Ascorbate and aldarate metabolism | 0.0007320 | 121.54 |
| Peroxisome | 0.00001966 | 117.28 |
| PPAR signalling pathway | 0.00002104 | 115.18 |
| Drug metabolism | 0.00001064 | 106.53 |
